# Enhanced Protein Photo‐Stability Analysis Using SRCD in the Presence of Phospholipid SUVs

**DOI:** 10.1002/chem.202500792

**Published:** 2025-06-08

**Authors:** Claudia Honisch, Martina Rotondo, Mario Monaco, Stefano Tartaggia, Rohanah Hussain, Giuliano Siligardi, Paolo Ruzza

**Affiliations:** ^1^ Institute of Biomolecular Chemistry of CNR Padua Unit via Marzolo,1 Padova 35131 Italy; ^2^ Department of Biology University of Naples Via Cinthia,26 Napoli 80126 Italy; ^3^ Department of Chemical Sciences University of Padua Via Marzolo, 1 Padova 35131 Italy; ^4^ Diamond Light Source Ltd Harwell Science and Innovation Campus Didcot Oxfordshire OX11 0DE United Kingdom

**Keywords:** circular dichroism spectroscopy, lipid‐protein interaction, lipoxidation, protein conformational stability, synchrotron radiation

## Abstract

The interaction between lipids and proteins impacts a multitude of cellular processes and may contribute to the onset of several pathologies and aging. Such processes are frequently linked to oxidative stress, whereby polyunsaturated fatty acids act as substrates for in vivo lipoxidation. The subsequent lipid peroxidation and/or isomerization is known to affect membrane organization, as well as to modify proteins and DNA, leading to functional alterations. The aim of this study was to evaluate the capacity of UV‐denaturation experiments to induce lipid modification and to investigate the influence of lipid presence on the conformational stability of selected soluble model proteins. The high photon flux and brilliance of the incident beam light of Diamond Light Source B23 for sinchrotron radiation circular dichroism (SRCD) was used to induce protein denaturation. This was acheived by scanning 30 repeated consecutive SRCD spectra in the far‐UV region that, being diagnostic of protein folding, enabled the estimation of the protein photostability. Our findings show that the presence of lipid vesicles (SUVs) significantly impacts the UV denaturation of proteins, preserving the native structure in proteins with a high helical content. This suggests that lipids may play a protective role against light‐induced damage to proteins.

## Graphical abstract

The influence of lipid presence on the photostability of proteins was assessed using a synchrotron radiation circular dichroism UV‐denaturation experiment at Diamond B23 beamline. The presence of SUVs preserves the native structure in proteins, particularly in those with a high helical content, suggesting that lipids may play a protective role against light‐induced damage to proteins.

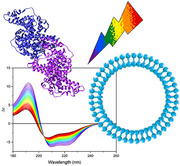

## 1 Introduction

The interaction of proteins with membranes is of great significance in the context of biological phenomena, as it plays a pivotal role in a multitude of biological processes. The interactions between lipids and proteins can destabilize the proteins’ native conformation, modifying protein‐protein interactions and triggering their aggregation.^[^ ^1^ ^]^ Moreover, membrane‐bound proteins have the capacity to modulate several key processes, including lipid lateral diffusion, membrane tension and fluidity, and lipid phase separation.^[^ ^2^ ^]^ It can be concluded that lipid‐protein interactions play a significant role in the biological functions of cells and can be involved in the development of diseases.^[^ ^3^ ^]^ In particular, it is generally agreed that there is a correlation between aging and the accumulation of oxidatively damaged proteins, lipids, and nucleic acids.^[^ ^4^ ^]^ Although the protein‐lipid interactions received more attention based on their biological role, the effect of oxidative stress on protein‐lipid interactions has been studied less.

**Scheme 1:**
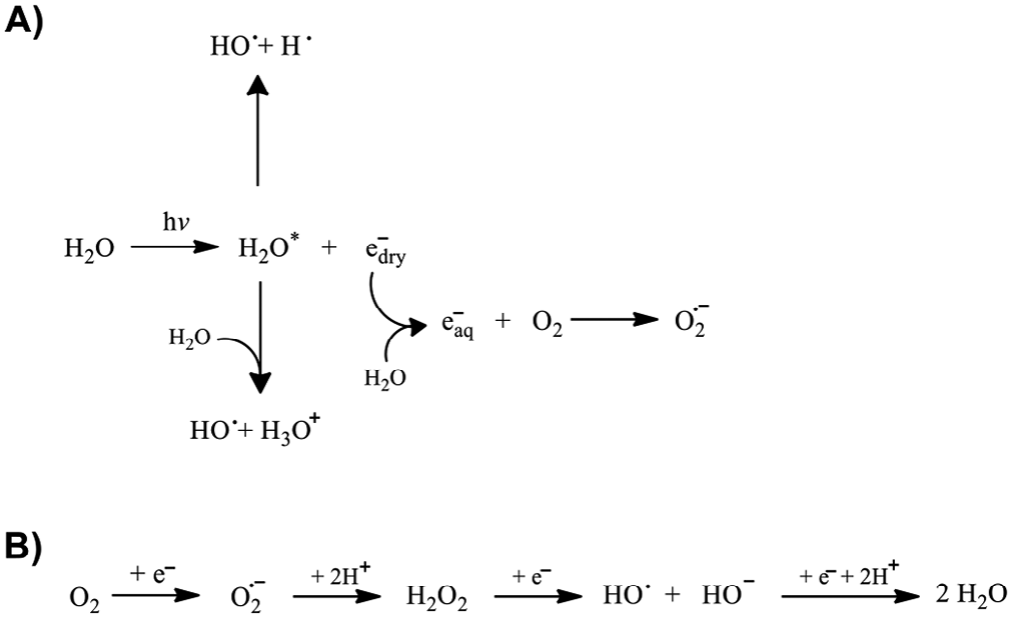
Proposed scheme of ROS production A) in vitro by water photolysis and B) in vivo inside cells.

Several biophysical and structural methods are used to study this biomolecular interaction.^[^ ^5^ ^]^ Of the available techniques, circular dichroism is the optimal method for characterizing and monitoring the folding of proteins in solution as a function of environmental factors, including lipids. The utilization of synchrotron radiation sources has been extended to lower wavelengths within the far‐UV region, thereby enhancing the signal‐to‐noise ratio in comparison to that achievable with a bench‐top CD spectropolarimeter.^[^ ^6^, ^7^ ^]^ The irradiation of high photon flux and high brilliance of synchrotron beam light of Diamond B23 beamline can induce a significant decrease in protein‐ordered structure when repetitive scans are recorded in the far‐UV region. As each spectrum is scanned in about 2.5 minutes, 30 repeated consecutive scans, corresponding to 75 minutes of irradiation in the far‐UV region, will enable the evaluation of the protein photostability.^[^ ^8^ ^]^ This is because the far‐UV irradiation can induce a protein conformational denaturation that is irradiation time‐dependent.^[^ ^8^ ^]^ In this study, the relative rate of UV protein denaturation was found to be significantly affected by ligand interactions, thus proving to be an invaluable method for determining binding interactions of ligands with negligible UV absorption, such as those observed with lipids.^[^ ^8^ ^]^ Of the various mechanisms proposed to elucidate this phenomenon, the involvement of free radicals and reactive oxygen species (ROS) is the most prominent.^[^ ^9^ ^]^ Indeed, in a dilute aqueous solution at micromolar concentrations, the radiation interacts exclusively with water (at a concentration of approximately 55 M), while the radiation effects on the solute are entirely indirect. The generation of reactive species by the photolysis of water molecules can be employed as a means of mimicking oxidative stress conditions. Indeed, during conditions of oxidative stress, ROS, including the superoxide anion (O_2_^•−^) and the hydroxyl radical (OH^•^), are generated (Scheme 1).

These species not only react with DNA or proteins but can also react with the phospholipids that constitute cell membranes, thereby causing their degradation. Lipids containing unsaturated fatty acids are highly susceptible to oxidant attack, and their free‐radical‐mediated oxidation, referred to as lipid peroxidation, has been linked to a number of deleterious effects.^[^ ^10^ ^]^ The process of lipid peroxidation generates a range of oxidation products, including highly reactive aldehydes and α,β‐unsaturated moieties that are capable of forming covalent adducts with biopolymers.^[^ ^11^ ^]^ Furthermore, at the end of the 20^th^ century, research began to focus on the role of free radicals in the isomerization of unsaturated fatty acids in aqueous solutions. Indeed, given that in eukaryotic cells the natural fatty acids predominantly possess the *cis* configuration, the formation of *trans* isomers can be regarded as a significant structural alteration in the context of cellular stress.^[^ ^12^ ^]^ Recently, there has been a growing interest in the biological effects related to these reactions. Indeed, lipid peroxidation and/or isomerization have been demonstrated to exert significant effects on membrane organization, and can modify proteins and DNA, leading to functional alterations. It has been postulated that both these processes may be involved in the aging and the pathogenesis of various diseases, including atherosclerosis, neurodegenerative disorders, autoimmune diseases, and type 2 diabetes.^[^ ^13^, ^14^, ^15^ ^]^

The objective of this study was to assess the potential of UV‐denaturation experiments to induce lipid modification and to investigate the impact of lipid presence on the conformational stability of selected soluble model proteins, including bovine serum albumin (BSA), hen egg white lysozyme (HEWL), ubiquitin (Ubi), and rabbit IgG (IgG). To this purpose, a series of UV‐denaturation experiments were conducted at the synchrotron beamline B23 of the Diamond Light Source in the presence of 1:1 mol/mol DMPG:DOPC small unilamellar vesicles (SUVs).

Our results show that the presence of SUVs strongly influenced the UV denaturation of protein, preserving the native structure in proteins characterized by the presence of a high amount of helical structure, suggesting a protective role of lipids toward light‐induced damage of proteins.

## 2 Results and Discussion

### 2.1 Effects of Lipids and Temperature on the Secondary Protein Structure

**Figure 1:**
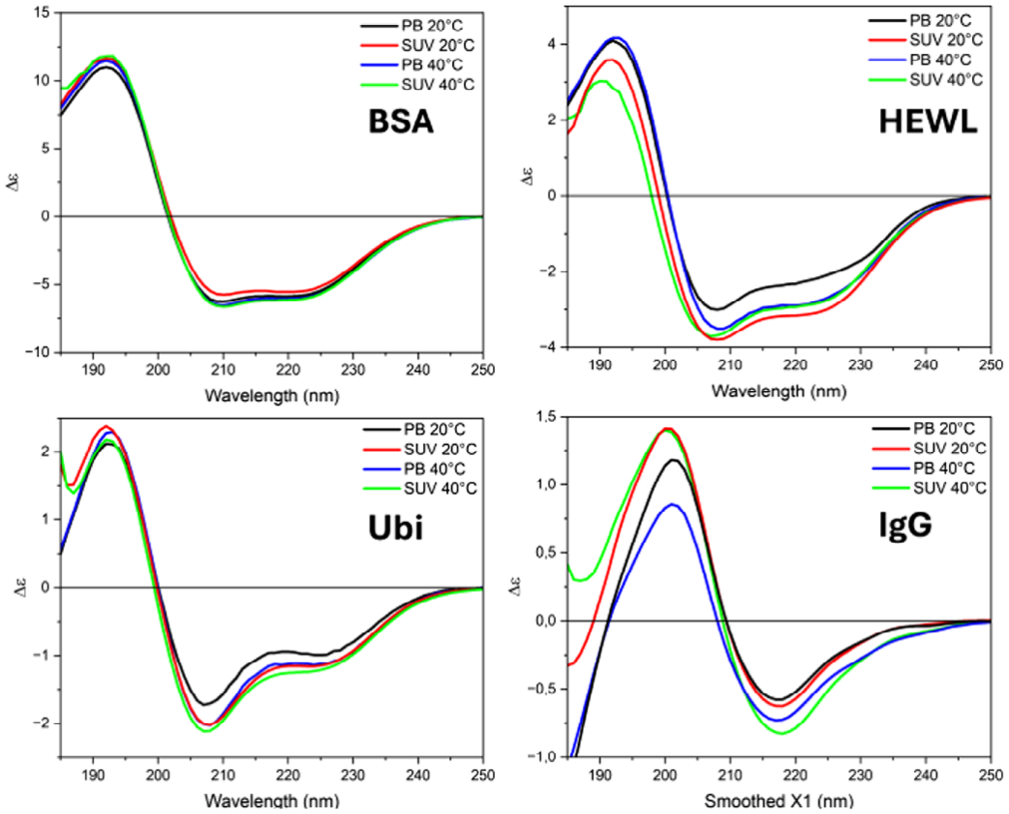
Far‐UV SRCD spectra of BSA, HEWL, Ubi, and IgG in PB and DMPG:DOPC SUVs (1:1 mol/mol) at 20 °C and 40 °C, respectively.

**Figure 2:**
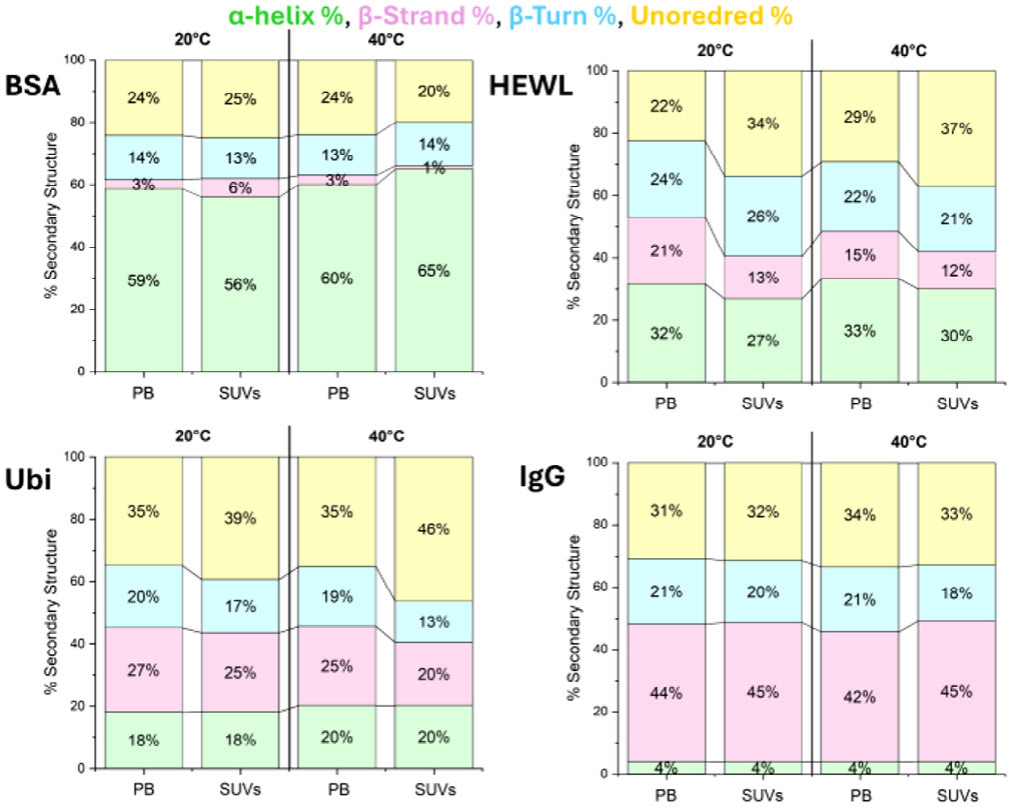
The percentage of the secondary structure content determined from SRCD data of BSA, HEWL, Ubi, and IgG in PB and SUV at 20 °C and 40 °C.

Four proteins, BSA, Ubi, HEWL, and IgG were studied by synchrotron radiation circular dichroism (SRCD) spectroscopy in phosphate buffer (PB) and in DMPG‐DOPC SUVs (1:1 mol/mol) (see preparation below in “CD spectra of DMPG:DOPC liposomes” section) at 20 °C and 40 °C, respectively (Figure 1). In terms of visual spectral changes, small changes were observed for all four proteins upon change in solvent or temperature. In particular, the smallest for BSA and Ubi, a little bit bigger for HEWL, and even bigger for IgG. However, these spectral changes are better evaluated in terms of the content of elements of secondary protein structure estimated from SRCD data, and their changes have been reported as a function of temperature and solvent environment in Figure 2.

For BSA (upper left panel of Figure 2), the protein folding is not significantly affected by the different solvent environments between PB and SUV, even if at 20 °C in the presence of SUVs the β‐strand content is increased (from 3% to 6%) at the expense of the α‐helix content (from 59% to 56%). Raising the temperature to 40 °C in the presence of SUVs results in an increase in the α‐helix content, at the expense of the unordered content.

For HEWL (upper right panel of Figure 2), at 20 °C there is an increase in unordered content (34%) in SUV compared to that in PB (22%) at the expense of both β‐strand and α‐helix content. At 40 °C, the unordered conformation is increased in PB (from 22% to 29%) at the expense of β‐strand, while in SUVs, the increase in unordered content is accompanied by a decrease in other secondary structures.

For Ubi (lower left panel of Figure 2), at 20 °C no significant solvent effect is observed in both α‐helix and β‐strand content, while the unordered conformation content increases from PB to SUVs. At 40 °C, a large increase in the unordered conformation is observed in SUV (46%), accompanied by a decrease in the β‐strand and β‐turn content.

For IgG (lower right panel of Figure 2), no significant effect on the protein secondary structure is observed when switching from PB to SUVs at 20 °C as also with the increase in temperature.

### 2.2 Effect of SUVs on Protein UV‐Denaturation

**Figure 3:**
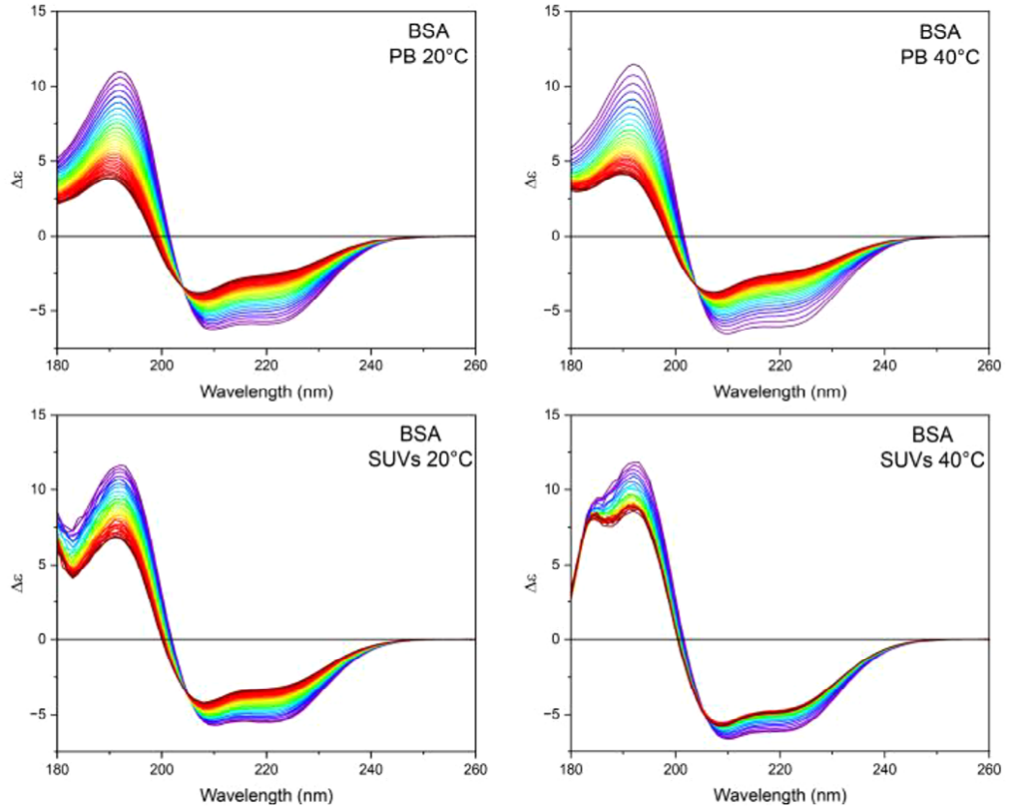
UV‐denaturation experiments of BSA. Thirty consecutive repeated scans of BSA (1.5 µM) either in 10 mM PB, pH 7.4, or in 1:1 mol/mol DMPG:DOPC SUVs (indicated). The spectra were acquired at 20 °C or at 40 °C (indicated) in the 185–250 nm range at the module A end station of beamline B23 at Diamond Light Source synchrotron facility, Harwell Science and Innovation Campus (Didcot, UK), using a 0.02 cm quartz cuvette. Bandwidth was 1 nm, scan speed was 39 nm/min, and top‐up mode ring current was 300 mA.

The impact of phospholipids on the stability of a selected protein was assessed through a protein UV‐denaturation assay, conducting 30 repeated consecutive SRCD scans of the protein solution in PB and DMPG:DOPC SUVs at 20 °C or 40 °C, respectively. For each protein, the BSA (Figure 3), HEWL, Ubi and IgG (Figures 1S, 2S and 3S in the Supplementary Material, respectively) spectra demonstrated a gradual spectral change in intensity magnitude and shape from the 1^st^ to the 30^th^ spectrum. These data indicate a progressive loss of the native structure of proteins, which is predominantly due to the action of ROS generated by the photolysis of water molecules.^[^ ^9^ ^]^ Aromatic amino acids, such as tryptophan and tyrosine, can also contribute to the generation of ROS.^[^ ^16^, ^17^ ^]^ Indeed, they can be photoexcited by synchrotron radiation, which induces the transfer of photoionisation electrons that produce ROS.^[^ ^18^, ^19^, ^20^ ^]^

A comparison of the spectra recorded in the two different environments reveals that the variation of the CD signal is more pronounced in the samples in PB than in the presence of SUVs.

The comparison between the experiments conducted at 20 °C and 40 °C demonstrated that an increase in temperature enhanced the protein denaturation induced by light irradiation in PB to a greater extent than in SUVs.

**Figure 4:**
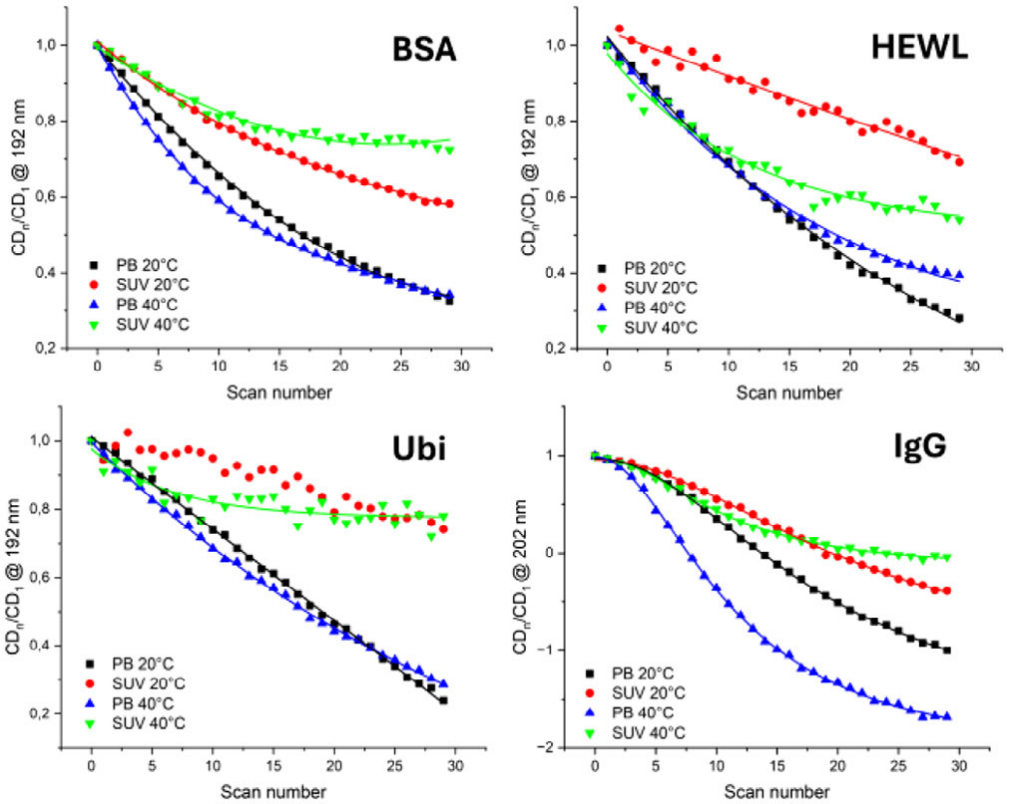
Time course of CD variation (CD_n_/CD_1_), with CD_1_ the values of the first scan at the maximum wavelength for each protein under each temperature and solvent condition as indicated, versus scan number.

The impact of UV denaturation on the secondary structure content of BSA, HEWL, Ubi, and IgG is illustrated in Figure 4. It can be observed that 30 consecutive repeated scans of the buffer solution at 20 °C induced a loss of native structure, which was greater for proteins with higher α‐helical content. The decrease of α‐helical content was accompanied by an increase in β‐strand and unordered structures. In contrast, the IgG protein having a high content of β‐strand structure showed a moderate decrease in its content, with an increase in unordered structure. Increasing the temperature (40 °C) reduces the loss of α‐helical structure after 30 scans for the HEWL and Ubi proteins, while the variation in the α‐helical content remains unchanged compared to the test conducted at 20 °C for the BSA protein.

Similarly, IgG protein also did not show any significant difference in secondary structure content at 40 °C compared to that at 20 °C.

**Figure 5:**
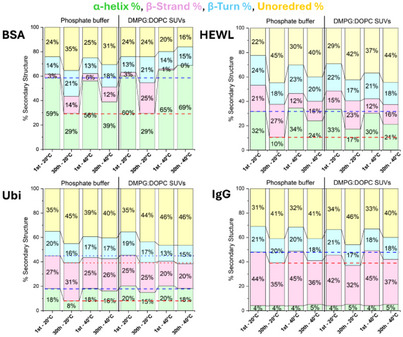
Secondary structure estimation of the analyzed proteins (indicated) either in 10 mM PB, pH 7.4, or in 1:1 mol/mol DMPG:DOPC SUVs, at the beginning (1^st^) and the end (30^th^) of the UV‐denaturation experiments at the two different temperatures (20 °C and 40 °C).

The presence of phospholipids has a clear effect on preserving the native structure after 30 consecutive scans analyzing the α‐helix content of each protein. This effect is amplified by heating, as can be observed in the case of the BSA protein, where an increase in the α‐helix content is observed at the expense of the β‐structure. In proteins rich in β‐structure (IgG), the presence of phospholipids has minimal effect on the secondary structure, as previously highlighted for the buffer solution heating. The analysis of the protein denaturation process can be obtained by plotting the ellipticity values at a selected wavelength against the scan number, which is proportional to the synchrotron irradiation exposure time, that for 30 consecutive repeated scans corresponded to 75 minutes (Figure 5).

The rate of protein photo denaturation (decay rate) was calculated by fitting the plots of ellipticity values at selected wavelengths versus the number of scans for each protein UV‐denaturation assay with a two‐phase exponential decay function. The fitting enabled the determination of the decay rate (*k* = 1/*t*) and half‐life (*t*_1/2_ = *t* ln2) of each component (Table 1). The Single Value Decomposition (SVD) analysis of the 30 UV‐denaturation SRCD spectra highlighted two components for each protein, and was carried out with the CDApps software.^[^ ^21^ ^]^

**Table 1:** Time constant (*t*) and decay rate (1/*t*) values of UV denaturation of proteins (indicated) in different environments and temperatures determined by acquiring 30 consecutive repeated scans.

	Time Constant [*t*]				Decay‐Rate [*k* = 1/*t*]			
	PB		SUV		PB		SUV	
Sample	20 °C	40 °C	20 °C	40 °C	20 °C	40 °C	20 °C	40 °C
HEWL	8.856	7.469	13.113	6.822	0.113	0.134	0.076	0.147
UBI	2.449	19.437	13.593	9.298	0.408	0.051	0.074	0.108
BSA	18.328	10.165	0.788	15.326	0.055	0.098	1.268	0.650
IgG	9.786	3.622	10.810	5.797	0.102	0.276	0.092	0.172

**Figure 6:**
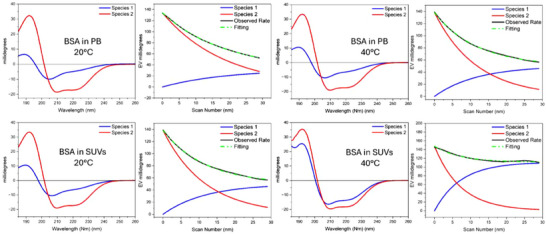
Calculated far‐UV CD spectra of the two SVD component species (red and blue lines) of the BSA UV‐denaturation assay in PB and SUV at 20 °C and 40 °C, respectively, and variation of their content of the two species compared to the observed rate (black) and the sum of the two species (green, dash dot‐dot) as the experiment progressed.

For BSA in PB and SUV at 20 °C and 40 °C, the two SVD component species are reported in Figure 6, whilst the corresponding SVD analysis for HEWL, Ubi, and IgG other experiments are reported in the Supplementary Material (Figures 4, 5, 6).

In summary, the order of stability to UV denaturation at 20 °C in PB was BSA > IgG ≥ HEWL > UBI, whereas at 40 °C, IgG was more susceptible compared to HEWL. In SUV at 20 °C however, except for BSA, all proteins were more resistant to UV denaturation with high time constants, but this order was reversed when they were incubated in 40 °C SUV, with BSA appearing to be more stable to radiation than Ubi, HEWL, and IgG.

The UV‐denaturation process follows first‐order kinetics, as shown in the SVD (Figure 6) for example, in BSA with 2 species from native to denatured structure as a function of UV exposure time with the observed and fitted rate of 0.054 s^−1^, which matches closely with the first exponential decay rate calculated in Table 1.

### 2.3 CD Spectra of DMPG:DOPC Liposomes

The preparation of small unilamellar liposomes was achieved through the extrusion of a 1:1 mol/mol DMPG:DOPC mixture. In pure methanol, the CD signal of the phospholipid mixture is minimal (see Figure 7S in the Supplementary Material). Conversely, the supramolecular organization of phospholipids within liposomes results in the formation of a broad band with a maximum around 220 nm, which can be attributed to the n→π* transition of the ester chromophore group. In the naturally occurring L‐enantiomer of phospholipids in liposomes, the band is consistently observed to be positive.

**Figure 7:**
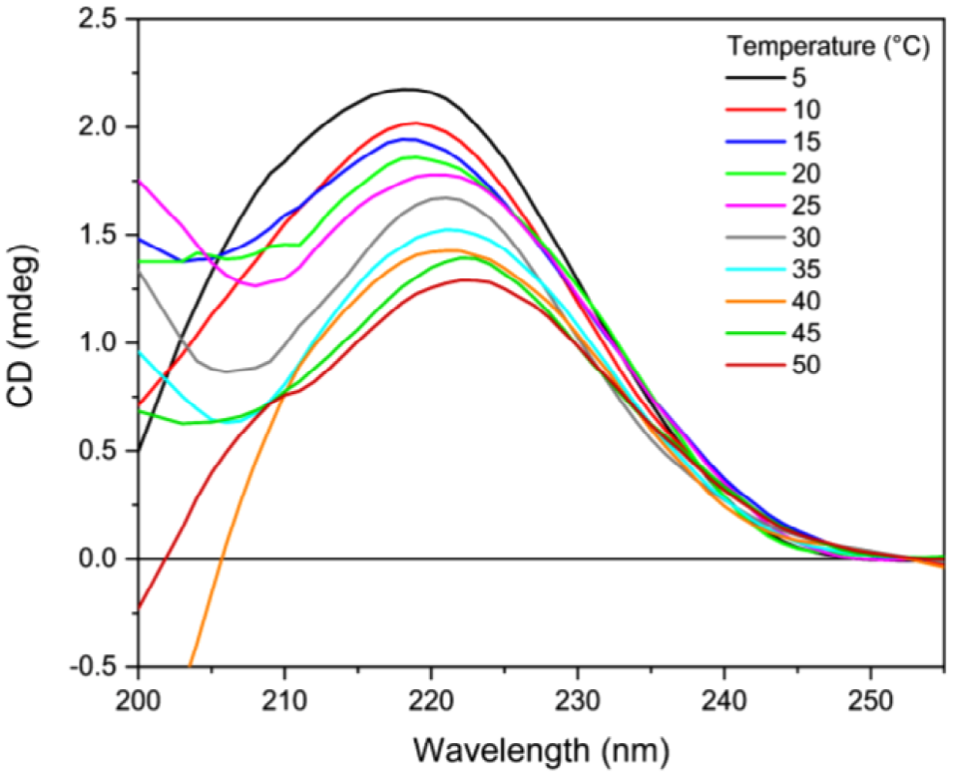
Far‐UV CD spectra of 1:1 mol/mol DMPG:DOPC SUVs recorded at different temperatures (indicated). Spectra were acquired in the 200–260 nm by a Jasco J‐1500 spectropolarimeter using a 0.1 cm quartz cuvette, bandwidth 1 nm, and scan speed 50 nm/min.

**Figure 8:**
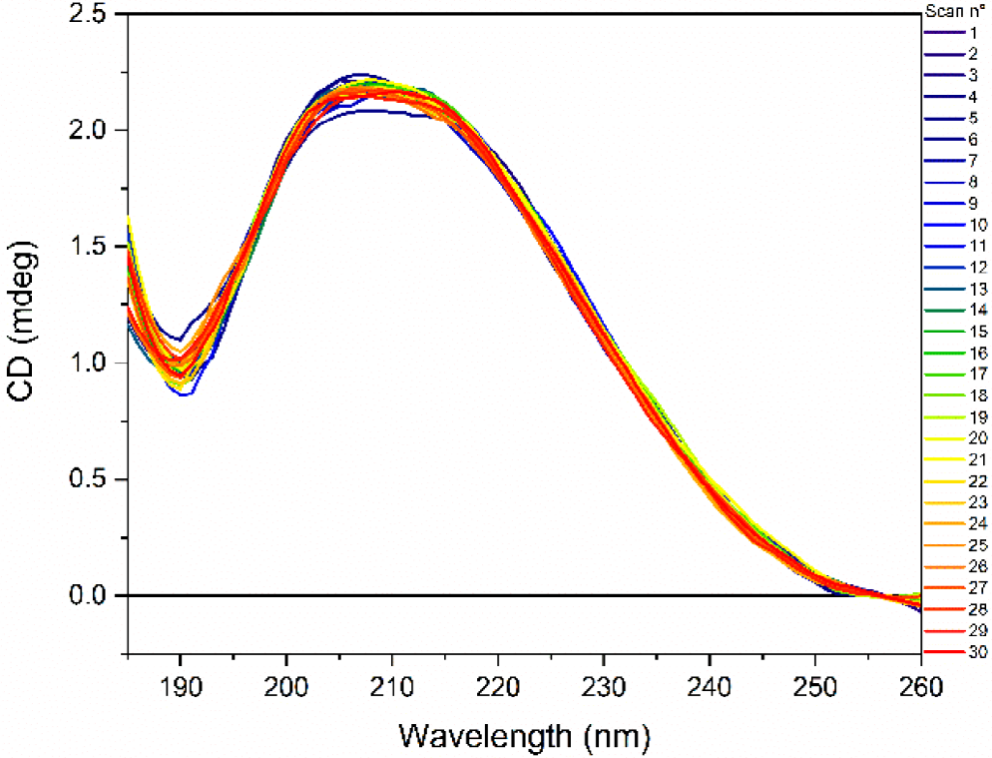
UV‐denaturation experiments of 1:1 mol/mol DMPG:DOPC SUVs. Thirty consecutive repeated scans were acquired either at 20 °C or at 40 °C in the 185–260 nm range at the module A end station of beamline B23 at Diamond Light Source synchrotron facility, Harwell Science and Innovation Campus (Didcot, UK), using a 0.02 cm quartz cuvette. Bandwidth was 1 nm, scan speed was 39 nm/min, and top‐up mode ring current was 300 mA.

As illustrated in Figure 7, both intensity and position of this band are directly correlated with temperature, exhibiting a greater intensity at temperatures below the phase transition temperature (Tc). At 5 °C, the CD band is centered at approximately 219 nm, with an intensity of roughly 2.18 mdeg. In this condition, the hydrocarbon chains of phospholipids adopt a *trans* orientation. Upon increasing the temperature, a shift toward higher wavelengths and a reduction in the intensity of the CD band have been observed (Figure 8S). At temperatures above the Tc, the hydrocarbon chains of phospholipids adopt either *gauche* or *trans* orientation.^[^ ^22^ ^]^ At 50 °C, the positive band is centered at 224 nm with an intensity of about 1.28 mdeg. These observations indicate that the reduction of the conformational flexibility in the glycerol moiety of phospholipids (from monomeric to liposomal and a decrease in the fluidity of vesicles by low temperature) is paralleled by a significant increase in the CD band of phospholipids. The Tc value could be determined by plotting either the intensity at a given wavelength or the maximum of the CD band as a function of temperature. The value determined for the DMPG:DOPC SUV in water was approximately 25 °C (see Figure 20S in the Supplementary Material).

The results of the UV‐denaturation experiments on DMPG:DOPC SUVs indicate a slight variation in the intensity of the positive CD band at approximately 220 nm of the ester chromophore as a function of the number of scans (Figures 8 and 9S in the Supplementary Material), suggesting that phospholipids are very stable to UV radiation. This stability is greater at 20 °C than at 40 °C, indicating that a greater fluidity of vesicles is conducive to the loss of native structure.

To evaluate the effect of exposure to consecutive repeat scans on the structure of phospholipids, GC‐MS analysis of methyl esters SUV hydrolysate has been performed. Results show that lipids were not degraded, but an isomerization of the double bond present in the DOPC was detected (Figure 10S in the Supplementary Material). Additionally, preliminary SDS‐PAGE experiments conducted on HSA samples that were previously exposed to the synchrotron radiation UV‐denaturation experiment showed that the protein remains intact, with only a very negligible amount of degraded proteins observed with the B23 UV‐denaturation assay (Figure 11S in the Supplementary Material).

## 3 Discussion

The experimental results demonstrate that the protein UV denaturation of soluble proteins was solvent and temperature‐sensitive. In this study, the secondary structure content of BSA, with a high percentage of α‐helical structure, was partially preserved after 30 consecutive repeated scans in UV‐denaturation assays by the presence of SUVs. In contrast, the effect of lipid SUVs was negligible in the case of the IgG protein, which is characterized by a predominant β‐structure. Heating also mitigated the loss of secondary structure in α‐helical‐containing proteins in both PB and SUVs. The effect observed in the presence of SUVs could be attributed to the interaction of lipids with proteins, which stabilizes the secondary structure of the proteins themselves. This interaction is facilitated by the presence of hydrophobic pockets in the protein that allow the establishment of hydrophobic interactions between non‐polar amino acid residues of the proteins and the fatty acid tail of the lipid.^[^ ^23^, ^24^ ^]^ Furthermore, interactions between charged amino acid residues of proteins and polar head groups of lipids are also involved in these interactions. For example, lysine and glutamic acid residues on the N‐terminus of α‐synuclein demonstrate a robust electrostatic interaction with head groups of phospholipids.^[^ ^25^ ^]^ On the other hand, the effect observed with heating is associated with an enhancement in protein mobility. The increase in protein mobility determines a reduction in the number of protein molecules present per unit of time in the volume of solution exposed to synchrotron radiation and therefore able to react with ROSs generated by water photolysis. This is consistent with an increased protein UV denaturation on decreasing the concentration (unpublished results).

Unexpectedly, the repeated scanning of lipid SUVs in the far‐UV region over a period of 30 scans did not result in any significant degradation of the unsaturated phospholipid tails, or the generation of highly reactive species, suggesting a relative stability of SUVs toward 30 repeated consecutive scans. Only a partial isomerization of the double bond in DOPC molecules was observed, which could affect the organization of the phospholipid membrane, although no significant changes in protein‐lipid interactions were observed.

As proteins are the major targets of ROS action, it is not surprising that an increased number of pathologies have been linked to damage in protein structure arising from oxidation. However, protein oxidation is not limited to human diseases; oxidative damage to proteins is also of major significance in the food industry, the quality of agricultural materials, and the quality and efficacy of medicines, particularly peptide and protein drugs, vaccines, and antibodies. Consequently, the ability to reduce and control protein oxidation would be beneficial.

Our findings suggest that the presence of phospholipids can preserve the secondary structure of proteins from the degrading action of ROSs. Consequently, phospholipid vesicles could be used to preserve and increase protein stability in different formulations. Recent work has shown that phospholipids can accelerate or decelerate protein aggregation, altering the toxicity of protein aggregates, in a manner that directly depends on the lipid structure. The importance of lipid composition will therefore need to be further investigated also in relation to the fact that there is a growing body of evidence that the lipid profile of the membrane changes with aging.

## 4 Conclusion

Although the proteins under investigation in this study are not membrane proteins, they are exposed to cell membranes with high concentrations of phospholipids under physiological conditions. Consequently, the effect of lipids on the secondary protein structure was investigated using SRCD spectroscopy. Furthermore, the impact of phospholipids on protein denaturation induced by free radicals and ROS was also assessed through the implementation of SRCD UV‐denaturation experiments.

While there is a substantial body of knowledge concerning light‐induced damage to proteins, relatively little is known about the influence of lipids on this phenomenon. The results of our experiments demonstrate that lipids exert a significant influence on the UV‐denaturation assay, preserving the secondary structure of proteins with a high content of α‐helix conformation. This observation suggests a protective role against ROS induced by the photolysis of water molecules induced by synchrotron radiation. This study provides evidence that formulations containing phospholipid vesicles may be a viable option for protecting peptides and proteins from the action of light‐induced ROS, particularly in the pharmaceutical and food industries.

## 5 Materials and Methods

Proteins were purchased from Sigma‐Aldrich (Milan, Italy) and used without any other treatment.

Protein stock solutions were prepared in water and then diluted to 0.5 mg/mL (which corresponds to 7.5 µM for BSA, 34.2 µM for HEWL, 3.3 µM for IgG, and 58.1 µM for Ubi) either in 10 mM PB, pH 7.4, or in 1:1 mol/mol DMPG:DOPC SUVs.

SUVs were prepared to disperse lyophilized DMPG and DOPC at an equimolar ratio in water (5 mg/mL), sonicated in a tip sonicator for 20 minutes (1.5 seconds on, 1.0 second off, ampl. 20%), and then extruded in a polycarbonate membrane (0.50 µm) using the Avanti Polar Lipid Extruder Kit (Avanti Research, Birmingham, AL).

Protein UV‐denaturation assays were conducted at the module A end station of beamline B23 at the Diamond Light Source synchrotron (Harwell Science and Innovation Campus, Didcot, UK), recording 30 consecutive SRCD spectra at either 20 °C or 40 °C in the 185–250 nm range, using 0.02 cm quartz cuvettes. Bandwidth was 1 nm, scan speed was 39 nm/min, and top‐up mode ring current was 300 mA. 75 minutes was the total time required to scan the 30 consecutive repeated spectra. CD data were analyzed using the CDApps suite of programs.^[^ ^21^ ^]^ The content of elements of protein secondary structure was calculated using the CONTINLL algorithm.^[^ ^26^ ^]^

Thermal denaturation of phospholipids (Figure 7) was performed using a Jasco J‐1500 CD spectropolarimeter in a 0.1 cm quartz cuvette, ramping from 5 °C to 50 °C at a 2 °C/min rate and acquiring a CD spectrum every 5 °C.

For GC‐MS analysis, irradiated lipid samples were extracted twice, first with methanol/chloroform 2:1 and thereafter with chloroform. The organic phases were reunited and dried under nitrogen flow. The residue was dissolved in toluene, and CH_3_ONa 0.5 M in MeOH was added. The reaction was conducted at 50 °C for 10 minutes, then the solution was neutralized using acetic acid in water. The lipidic fraction was extracted three times in *n*‐hexane and dried over Na_2_SO_4_. The organic layer was separated by filtration and dried under N_2_ flow. The residue was dissolved into 1 mL *n*‐hexane and injected in an Agilent 6850 Network GC System (Agilent Technologies, Santa Clara, CA, USA) equipped with an Agilent 5975 Series MSD detection system. 1 µL of sample was injected in splitless mode (250 °C) and separated using a HP‐5MS 5% Phenyl Methyl capillary column (30 m × 250 µm). The oven temperature varied as follows: The Starting temperature was 80 °C, stayed so for 3 minutes, and then raised at 5 °C/min up to 250 °C, with a final stay at 250 °C for 5 minutes. Helium at 14.5 mL/min was the carrier gas. The detector's working temperature was 280 °C. The working range of the mass spectrometer was *m/z* 50–550.

## Conflict of Interest

The authors declare no conflict of interest.

## Supporting information



Supporting Information

## Data Availability

The data that support the findings of this study are available in the supplementary material of this article.
