# Supplementary material for: Enhanced Protein Photo‐Stability Analysis Using SRCD in the Presence of Phospholipid SUVs
Source: Chemistry. 2025 Jun 8;31(37):e202500792. doi: 10.1002/chem.202500792 (PMC12223356; doi:10.1002/chem.202500792)
Supplement: Supplementary file 1 — Supporting Information [file CHEM-31-e202500792-s001.docx]

Enhanced Protein Photo-Stability Analysis Using SRCD in the Presence of Phospholipid SUVs.

Claudia Honisch^# [a]^, Martina Rotondo^#[a,b]^, Mario Monaco^[a,c]^, Stefano Tartaggia^[a]^, Rohanah Hussain^[d]^, Giuliano Siligardi*^[d]^ and Paolo Ruzza*^[a]^

# These authors contributed equally to this work

[a] Institute of Biomolecular Chemistry of CNR, Padua Unit, via Marzolo,1, 35131, Padova, Italy

[b] Department of Biology, University of Naples, Via Cinthia,26, 80126, Napoli, Italy

[c] Department of Chemical Sciences, University of Padua, Via Marzolo, 1, 35131, Padova, Italy

[d] Diamond Light Source Ltd, Harwell Science and Innovation Campus, Didcot, Oxfordshire, OX11 0DE, United Kingdom

**SUPPLEMENTARY MATERIAL**


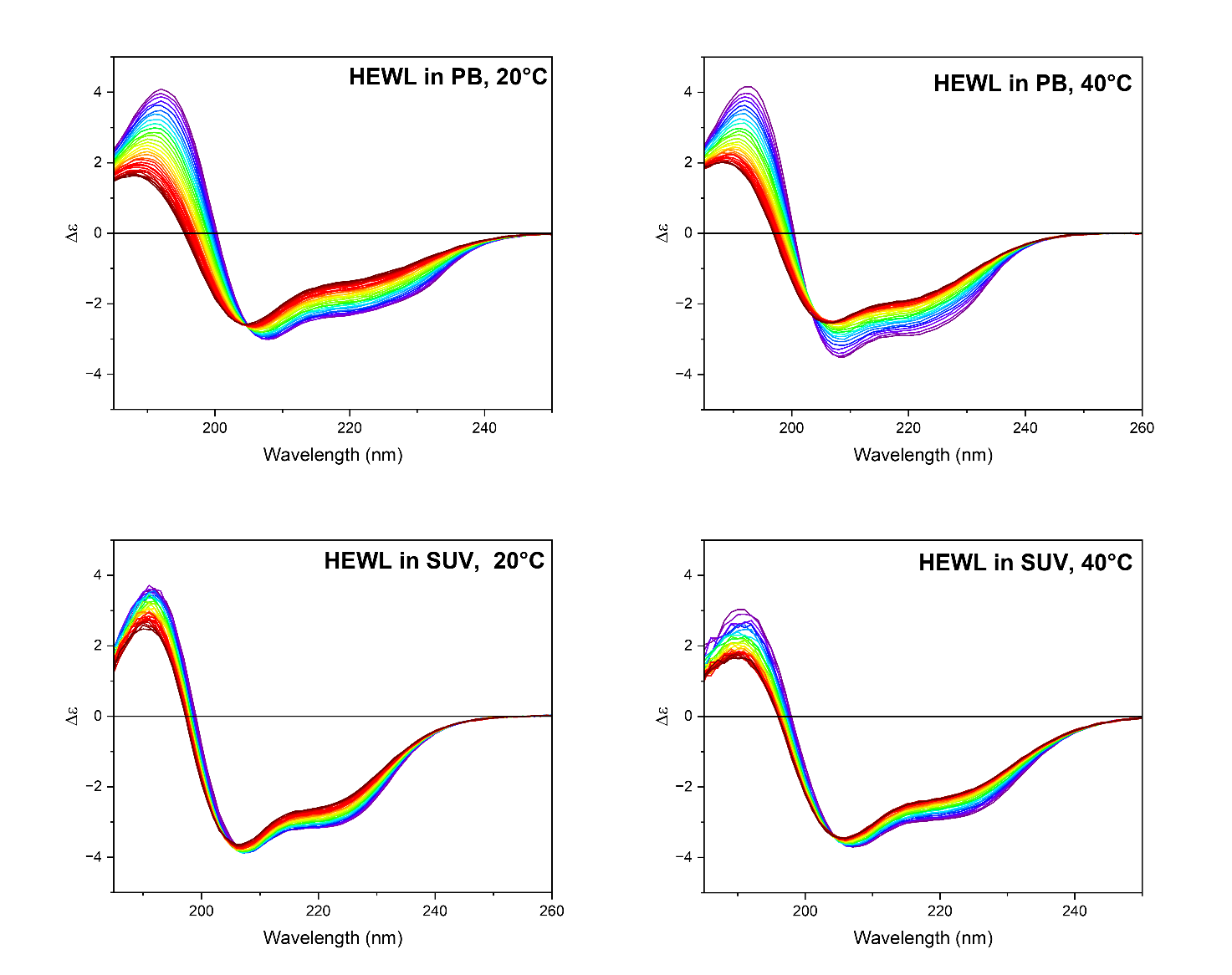


**Figure S1.** UV-denaturation experiments of HEWL protein. Thirty consecutive repeated scans of HEWL (34.2 µM) either in 10 mM phosphate buffer, pH 7.4, or in 1:1 mol/mol DMPG:DOPC SUVs. The spectra were acquired at 20°C or at 40°C in the 185-250 nm range at module A end station of beamline B23 at Diamond Light Source synchrotron facility, Harwell Science and Innovation Campus (Didcot, UK), using a 0.02 cm quartz cuvettes. Bandwidth was 1 nm, scan speed was 39 nm/min, and top-up mode ring current of 300 mA.


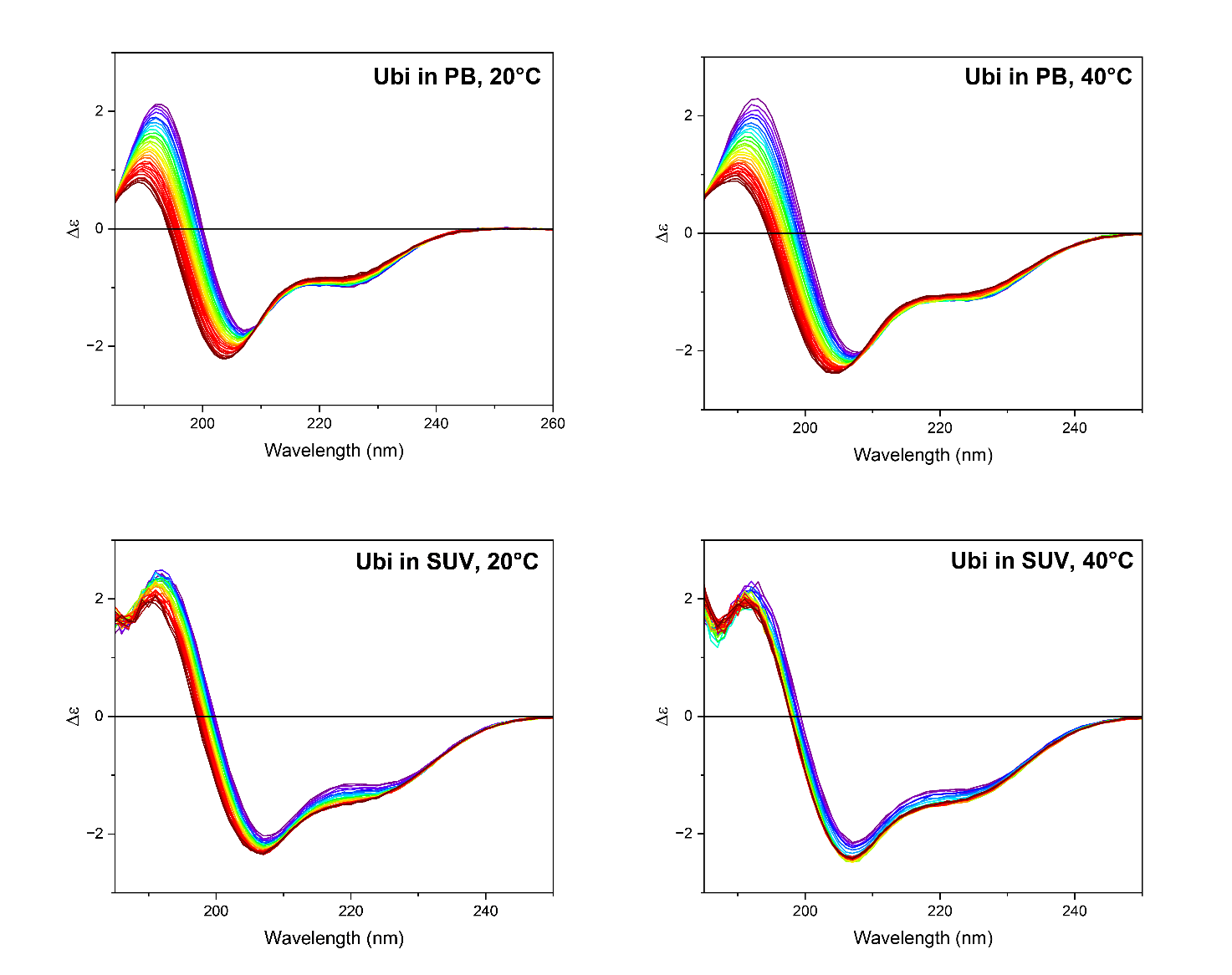


**Figure S2.** UV-denaturation experiments of Ubi protein. Thirty consecutive repeated scans of Ubi (58.1 µM) either in 10 mM phosphate buffer, pH 7.4, or in 1:1 mol/mol DMPG:DOPC SUVs. The spectra were acquired at 20°C or at 40°C in the 185-250 nm range at module A end station of beamline B23 at Diamond Light Source synchrotron facility, Harwell Science and Innovation Campus (Didcot, UK), using a 0.02 cm quartz cuvettes. Bandwidth was 1 nm, scan speed was 39 nm/min, and top-up mode ring current of 300 mA.


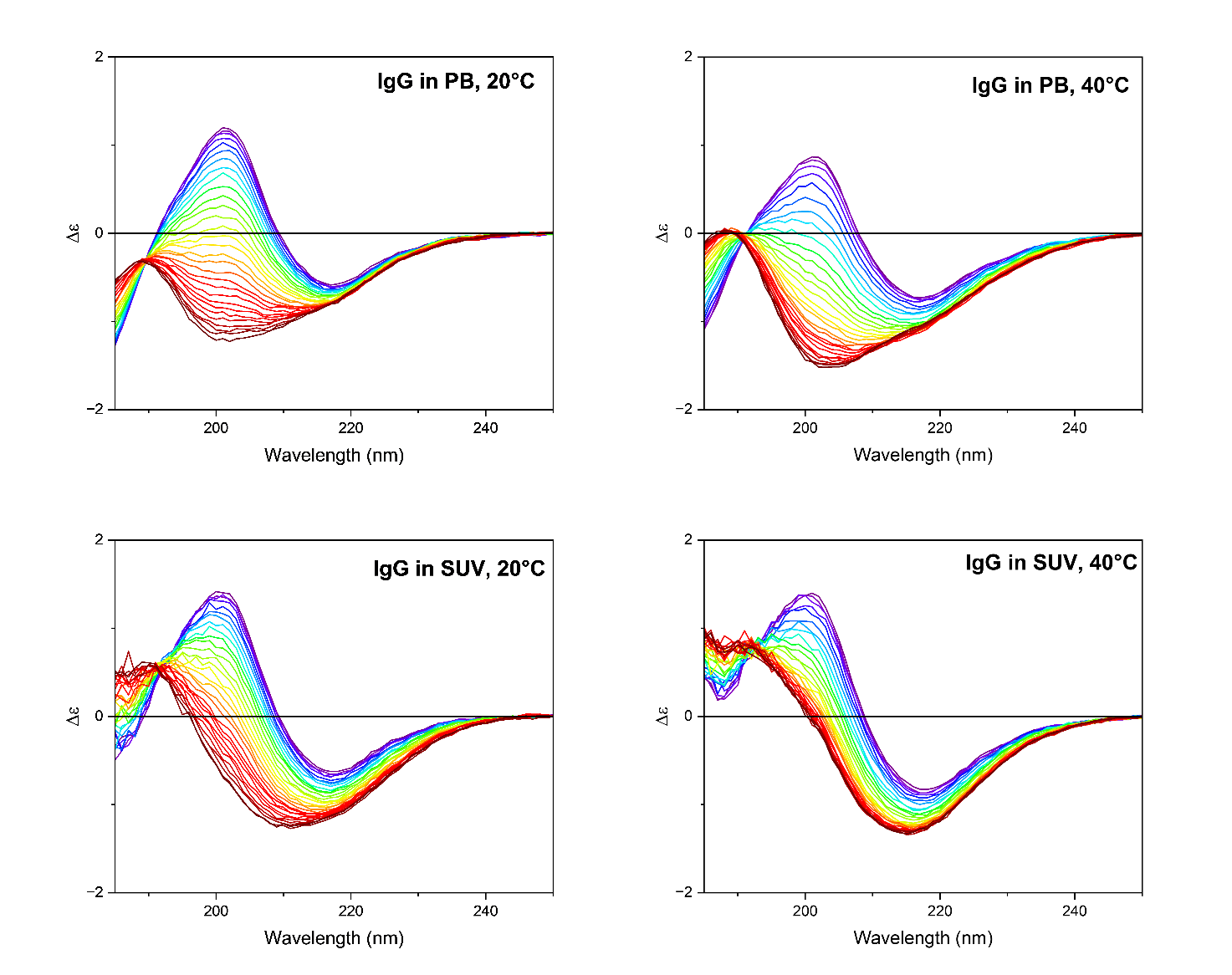


**Figure S3.** UV-denaturation experiments of IgG protein. Thirty consecutive repeated scans of IgG (3.3 µM) either in 10 mM phosphate buffer, pH 7.4, or in 1:1 mol/mol DMPG:DOPC SUVs. The spectra were acquired at 20°C or at 40°C in the 185-250 nm range at module A end station of beamline B23 at Diamond Light Source synchrotron facility, Harwell Science and Innovation Campus (Didcot, UK), using a 0.02 cm quartz cuvettes. Bandwidth was 1 nm, scan speed was 39 nm/min, and top-up mode ring current of 300 mA.


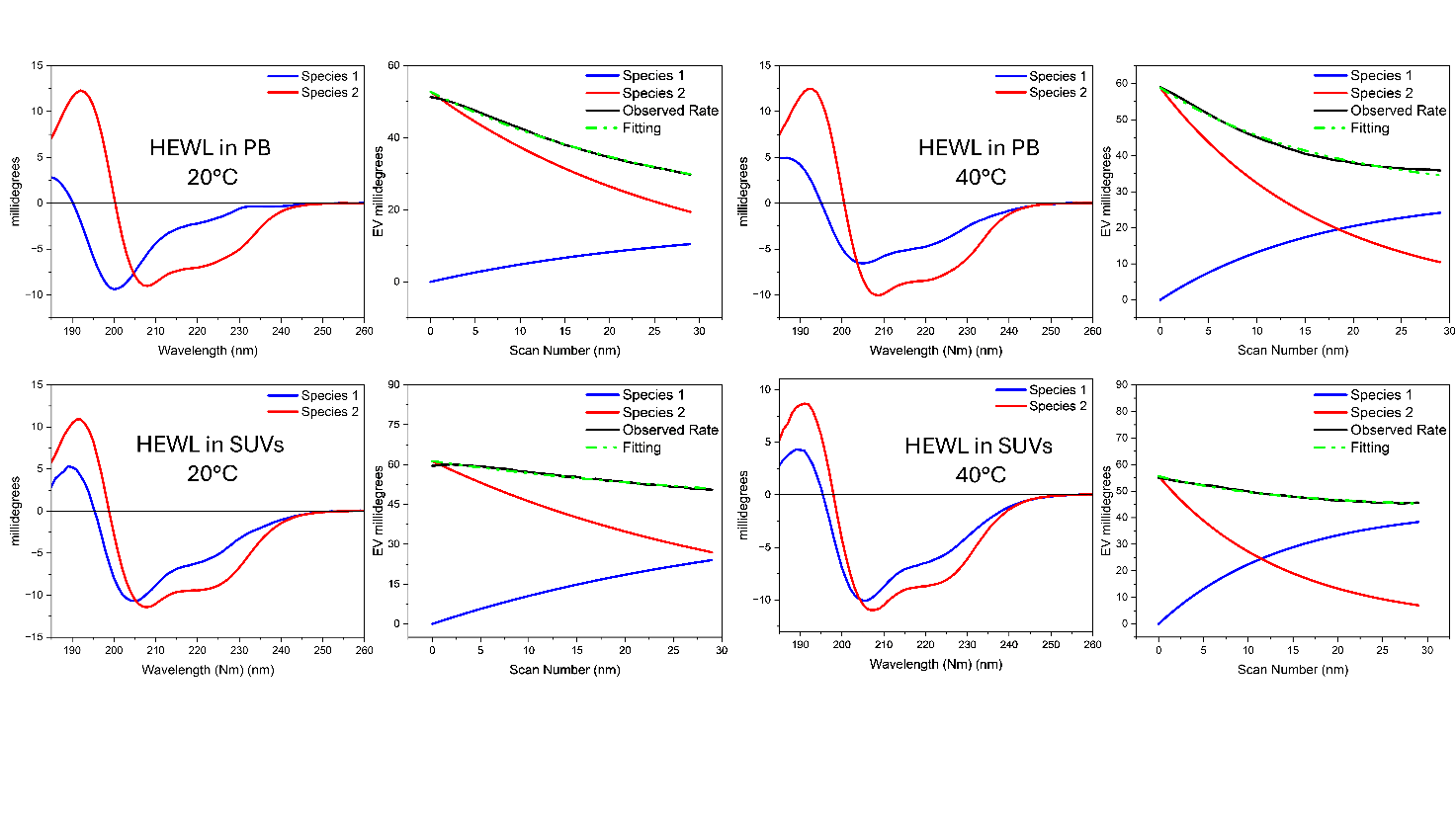


**Figure S4.** Calculated far-UV CD spectra of the two components determined by analysing the UV-denaturation data of HEWL in PB and SUV at 20ºC and 40ºC respectively, and variation in their content during the experiments.


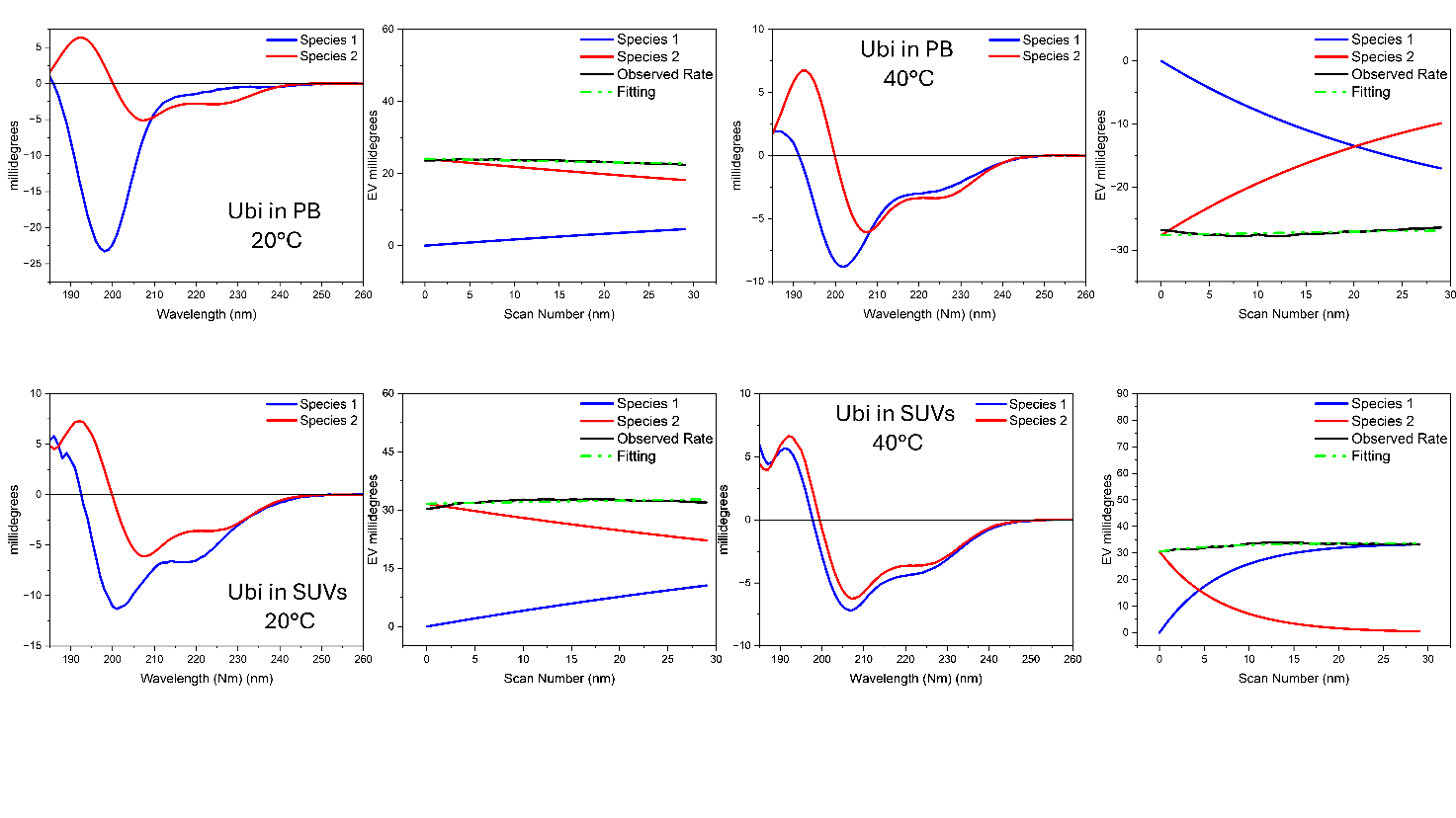
**Figure S5.** Calculated far-UV CD spectra of the two components determined by analysing the UV-denaturation data of Ubi in PB and SUV at 20ºC and 40ºC respectively, and variation in their content during the experiments.

**
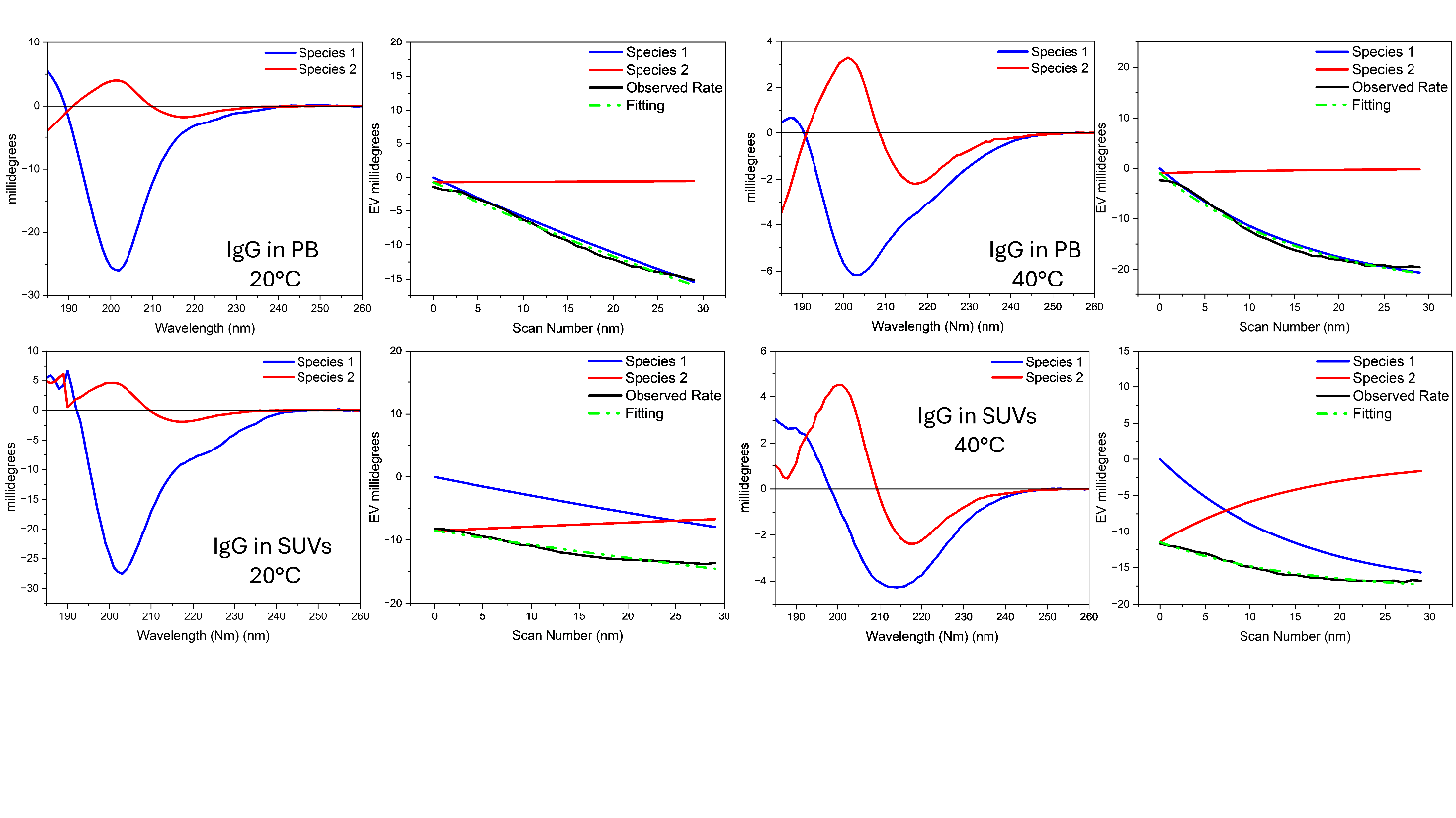
Figure** **S6.** Calculated far-UV CD spectra of the two components determined by analysing the UV-denaturation data of IgG in PB and SUV at 20ºC and 40ºC respectively, and variation in their content during the experiments.

**Figure S7.** Far-UV CD spectra of 1:1 mol/mol DMPG:DOPC in MeOH (0.5 mg/mL). Spectra were acquired in the 195-260 nm by a Jasco 1500 spectropolarimeter using a 0.1 cm quartz cuvette, bandwidth 1 nm, scan speed 50 nm/min.

**Figure S8.** Dependence of the CD l intensity magnitude of 1:1 mol/mol DMPG:DOPC SUVs as a function of temperature.

**Figure S9.** Time-course of CD values at 190 nm versus scan number of 1:1 mol/mol DMPG:DOPC SUVs . ◾ at 20°C; □ at 40°C.


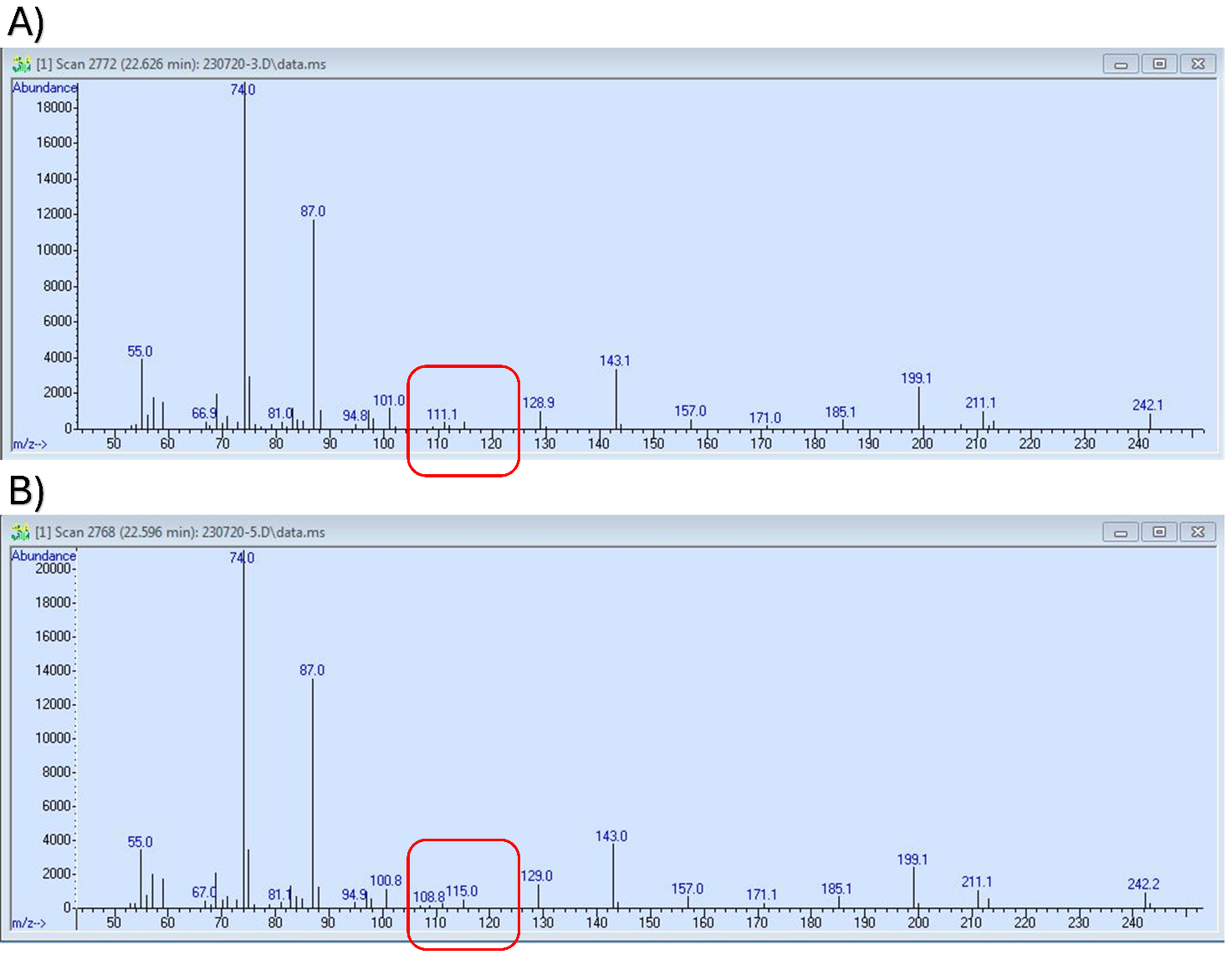


**Figure S10.** MS spectrum of the oleic acid present in SUV A) prior to, or B) after UV-denaturation experiment. Database of GC-MS instrument identifies the compound as *trans*-octadecenoic acid, methyl ester. The minor discrepancies between the two spectra are emphasised by a red box.


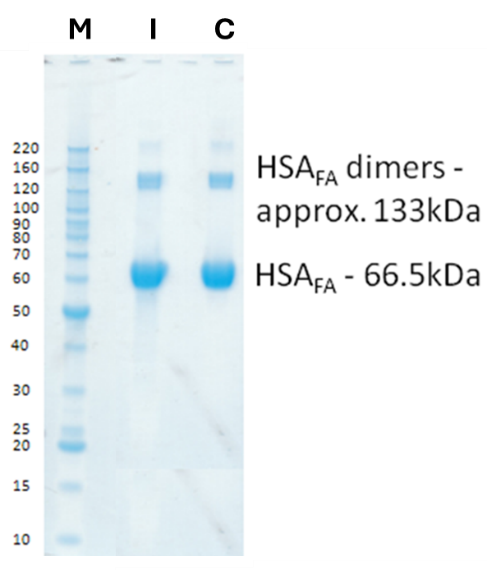


**Figure S11.** SDS-PAGE gel of UV irradiated HSA_fa_ of 50 consecutive repeated scans (I) compared to a non-irradiated control (C) against Invitrogen benchmark markers (M).
